# Multiple cellobiohydrolases and cellobiose phosphorylases cooperate in the ruminal bacterium *Ruminococcus albus* 8 to degrade cellooligosaccharides

**DOI:** 10.1038/srep35342

**Published:** 2016-10-17

**Authors:** Saravanan Devendran, Ahmed M. Abdel-Hamid, Anton F. Evans, Michael Iakiviak, In Hyuk Kwon, Roderick I. Mackie, Isaac Cann

**Affiliations:** 1 Energy Biosciences Institute, University of Illinois at Urbana-Champaign, Urbana, Illinois 61801, USA; 2 Carl R. Woese Institute for Genomic Biology, University of Illinois at Urbana-Champaign, Urbana, Illinois 61801, USA; 3 School of Molecular and Cellular Biology, University of Illinois at Urbana-Champaign, Urbana, Illinois 61801, USA; 4 Department of Animal Sciences, University of Illinois at Urbana-Champaign, Urbana, Illinois 61801, USA; 5 Department of Microbiology, University of Illinois at Urbana-Champaign, Urbana, Illinois 61801, USA

## Abstract

Digestion of plant cell wall polysaccharides is important in energy capture in the gastrointestinal tract of many herbivorous and omnivorous mammals, including humans and ruminants. The members of the genus *Ruminococcus* are found in both the ruminant and human gastrointestinal tract, where they show versatility in degrading both hemicellulose and cellulose. The available genome sequence of *Ruminococcus albus* 8, a common inhabitant of the cow rumen, alludes to a bacterium well-endowed with genes that target degradation of various plant cell wall components. The mechanisms by which *R. albus* 8 employs to degrade these recalcitrant materials are, however, not clearly understood. In this report, we demonstrate that *R. albus* 8 elaborates multiple cellobiohydrolases with multi-modular architectures that overall enhance the catalytic activity and versatility of the enzymes. Furthermore, our analyses show that two cellobiose phosphorylases encoded by *R. albus* 8 can function synergistically with a cognate cellobiohydrolase and endoglucanase to completely release, from a cellulosic substrate, glucose which can then be fermented by the bacterium for production of energy and cellular building blocks. We further use transcriptomic analysis to confirm the over-expression of the biochemically characterized enzymes during growth of the bacterium on cellulosic substrates compared to cellobiose.

The utilization of lignocellulosic biomass for production of liquid fuels is an important and promising alternative energy production. The structural polysaccharides of plant cell walls, i.e., cellulose and hemicellulose, represent a major source of fermentable sugars for potential production of biochemicals and biofuels^1^. However, the high cost and low enzymatic activities of cellulases, which are the enzymes responsible for converting pretreated biomass into fermentable sugars, are major challenges to biomass biorefineries^2^,^3^.

Cellulose, which accounts for a major proportion of the plant cell wall, consists of linear chains of β-1,4 linked glucose monomers. Crystalline cellulose is highly recalcitrant due to a high degree of crosslinking formed by hydrogen bonds, whereas amorphous cellulose is less rigid and more easily accessible to enzymatic degradation^4^. In order to degrade cellulose, different classes of cellulolytic enzymes function synergistically to release the glucose monomers, and these enzymes include endoglucanases, exoglucanases, and β-glucosidases. Endoglucanases (EC 3.2.1.4) randomly cleave internal regions of cellulose, producing shorter chains and also new chain ends. Exoglucanases (EC 3.2.1.91) bind to the reducing or non-reducing ends and processively release cellobiose, the repeating units in cellulose, as the major end product. Finally, β-glucosidases (EC 3.2.1.21) catalyze the conversion of cellobiose into glucose. The synergistic activity of these enzymes on cellulose degradation is reported elsewhere in details^4^,^5^. Some microorganisms encode genes for cellobiose phosphorylases, which use phosphate to cleave cellobiose, releasing glucose and glucose-1-phosphate in an energy conserving step^6^,^7^.

Fibrolytic bacteria and fungi use three predominant mechanisms to degrade recalcitrant plant cell wall polysaccharides, namely a free enzyme system, a cellulosomal system, and a Polysaccharide Utilization Loci system (PULs). In the free enzyme system, proteins are secreted and diffuse independently of one another and work synergistically to degrade plant cell wall polysaccharides^8^. For example, the model filamentous fungus *Trichoderma reesei* secretes an effective cocktail of free carbohydrate-active enzymes to degrade both cellulose and hemicellulose^9^. In contrast, cellulosomes are multi-enzyme complexes produced by certain anaerobic microorganisms, such as *Clostridium thermocellum*, *Clostridium cellulolyticum*, *Ruminoccocus flavefaciens* and *Ruminococcus champanellensis*, for efficient and ordered degradation of plant cell wall polysaccharides^10^,^11^,^12^,^13^,^14^. In the cellulosomes, multiple cellulolytic and hemicellulolytic enzymes are physically linked to a tethering protein scaffold, termed scaffoldin. Both catalytic domains and non-catalytic accessory modules are linked to facilitate binding to the substrate and are active against a variety of substrates such as cellulose, xylan, and pectin^10^. In a recently unraveled polysaccharide degradation strategy, it has been shown that members of the Bacteroidetes phylum, including several complex polysaccharide degrading *Bacteroides* spp and *Prevotella* spp, have evolved a polysaccharide deconstruction strategy where the genes encoding a complex network or machinery of sensors, transporters, and degradative enzymes are all clustered in a genome, an example being the xylan utilization system or XUS^15^.

Glycoside hydrolases (GH), the enzymes responsible for the cleavage of glycosydic bonds are grouped into families on the basis of amino acid sequence in the Carbohydrate Active enZYme (CAZY) database^16^,^17^. These enzymes have different structural folds, different mechanisms, and a wide range of substrates^16^,^18^. Currently, 44 of the 115 glycoside hydrolase families have been reported to contribute to plant cell wall degradation^19^. Some of the major glycoside hydrolases that are involved in cellulose degradation are GH1, GH3, GH5, GH6, GH7, GH8, GH9, GH12, GH44, GH45, GH48, GH61,GH74, and GH94^18^,^19^. Among them, GH family 9 is the second largest cellulase family and contains both endoglucanases and processive endoglucanases with varying domain architectures^11^,^12^,^20^. Most of the GH9 family enzymes have low or no activity on crystalline cellulose, but have activity on soluble cellulose derivatives, including carboxymethyl cellulose and phosphoric acid swollen cellulose^21^,^22^.

The rumen is home to a consortium of microorganisms that have evolved to efficiently degrade and ferment plant cell wall polysaccharides^23^,^24^. The primary organisms responsible for the degradation of plant cell walls are *Fibrobacter succinogenes*, *R. flavefaciens* and *R. albus*^25^. Specifically, *R. albus* 8 is a gram positive, fibrolytic, anaerobic bacterium and appears to be one of the most active cellulolytic bacteria in the rumen, with the capacity to degrade both cellulose and hemicellulose^26^,^27^. Although *R. albus* 8 has not been shown to produce a cellulosomal complex, related species *R. flavefaciens* and *R. champanellensis*, have been described to possess the capacity to synthesize a cellulosome^14^.

In the present report, we analyzed the draft genome sequence of *R. albus* 8 and identified a total of 22 genes encoding different glycoside hydrolases including 17 putative endoglucanases (GH5), 3 putative cellobiohydrolases (GH9) and 2 putative cellobiose phosphorylases (GH94), that may have roles in cellulose degradation. To explore the mechanism of cellulose hydrolysis utilized by this bacterium, we have undertaken a systematic approach to characterize the function of each of the predicted cellulose targeting genes. Here, the genes encoding the putative cellobiohydrolases, i.e., *ra3055*, *ra1589*, and *ra1743*, along with the putative cellobiose phosphorylases, *ra2122* and *ra2664*, were heterologously expressed and biochemically characterized. In order to further assess the roles of each domain within the polypeptide with the most active cellobiohydrolase activity or Ra3055, truncational variants of the polypeptide were expressed and biochemically characterized. Furthermore, synergistic interactions of an endoglucanase with an exoglucanase and the cellobiose phosphorylases were explored to gain insight into the complete cellulose deconstruction mechanism of *R. albus* 8.

## Results

### Cloning and expression of cellobiohydrolases from *R. albus* 8

**Figure 1:**
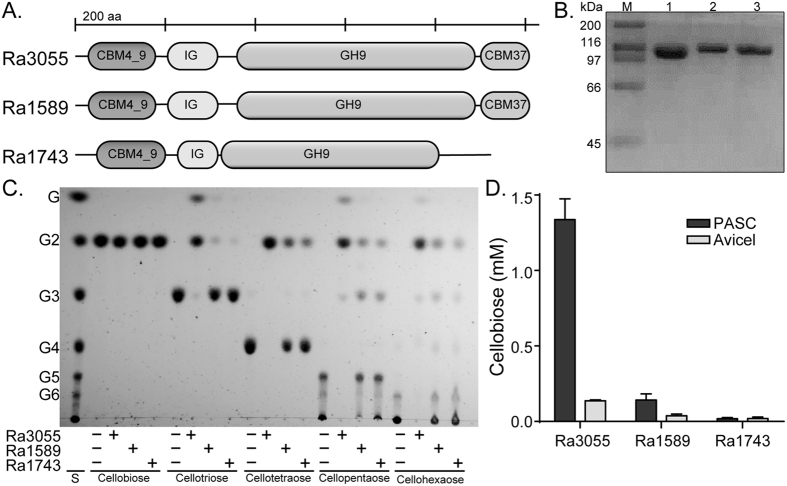
Three putative cellobiohydrolases of *Ruminococcus albus* 8. (**A**) Domain architecture were predicted using Pfam (http://pfam.sanger.ac.uk). (**B**) SDS-PAGE of the purified putative cellobiohydrolases. Lane M, molecular mass marker; Lane 1, Ra3055; Lane 2, Ra1589; Lane 3, Ra1743. (**C**) Thin layer chromatography for assessment of the hydrolytic activity of the putative cellobiohydrolases against β-1,4 linked cello-oligosaccharides. Each cellobiohydrolase (0.5 *μ*M) was incubated with cello-oligosaccharides (G_2_–G_6_) (0.5% wt/v) at 37 °C for 16 hours in sodium phosphate buffer (pH 6.5). The products were resolved and then visualized with methanolic orcinol and heating for 10 min at 80 °C. (**D**) Cellobiose release by the putative cellobiohydrolases on Avicel and PASC (0.5%). Each protein (0.5 μM) was incubated with the substrate (0.5% wt/v) in sodium phosphate buffer (pH 6.5) at 37 °C for 16 hours. The soluble sugars were seperated by high performance anion exchange chromatography (HPAEC) and detected using a pulsed amperometric detector (PAD). The concentration of the cellobiose was quantified by comparison of the peak area to a standard curve derived from known concentrations of cellobiose. Abbreviations: CBM, carbohydrate binding module; Ig, immunoglobulin-like module, GH9, glycoside hydrolyase family 9.

In an earlier report^28^, we discovered that seven endoglucanases from *R. albus* 8 hydrolyze cellulosic substrates to oligosaccharides, including cellobiose and cellotriose, with a far less proportion of glucose. Among these enzymes, endoglucanase Ra0903 was the most active on PASC^28^. To determine which genes the bacterium likely uses in addition to the endoglucanases to completely depolymerize cellulose, we used a bioinformatics approach to identify genes encoding potential enzymes that may further hydrolyze the end products of the *R. albus* 8 endoglucanases to fermentable sugars. Three genes encoding GH9 modules, likely functioning as cellobiohydrolases, were identified. The modular organization of the three gene products (Ra3055, Ra1589, and Ra1743) are presented in Fig. 1A. Both Ra3055 and Ra1589 are made up of a CBM4_9, an Ig-like motif, and a GH9 catalytic domain followed by a C-terminally located CBM37. The modular architecture of Ra1743 is not different from that of Ra3055 and Ra1589, except for the absence of the CBM37. Each gene product was purified to near homogeneity (Fig. 1B) and tested for the capacity to hydrolyze a number of cello-oligosaccharides. None of the polypeptides could hydrolyze cellobiose (or glucose with a degree of polymerization-DP- of 2). In contrast, all three polypeptides released cellobiose from cellotriose (DP3), cellotetraose (DP4), cellopentaose (DP5), and cellohexaose (DP6). The enzymatic activity of Ra3055 was the most active on each of the substrates, and in some cases (DP3, DP5 and DP6), the enzyme released glucose in addition to cellobiose (Fig. 1C). For a cellobiohydrolase, this was expected as cleavage of a cello-oligosaccharide with an odd number of degree of polymerization (DP) will leave a glucose unit. The enzymes were also tested for activity on amorphous cellulose, i.e., phosphoric acid swollen cellulose (PASC) and the model crystalline cellulose Avicel. Here also, Ra3055 showed a far superior activity compared with the other two proteins. In each case the enzymes released more reducing ends on PASC than Avicel. Based on the hydrolytic activity of the polypeptides on the substrates and their end products, we have assigned cellobiohydrolase activities to all three enzymes, with Ra3055 as the most active functional homolog.

### The pH and temperature optima of the cellobiohydrolases of *R. albus* 8

**Figure 2:**
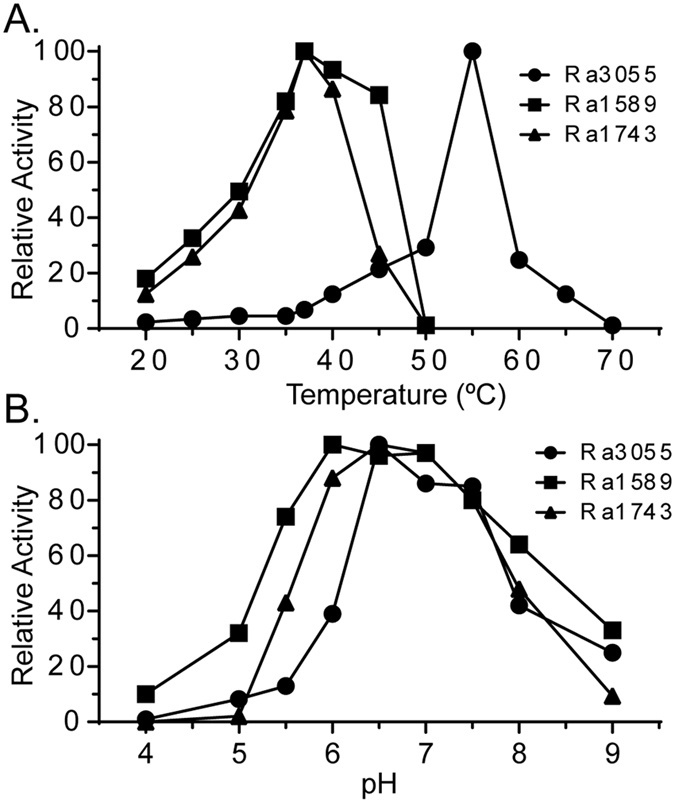
Temperature and pH profiles of three putative cellobiohydrolases of *R. albus* 8. (**A**) Each cellobiohydrolase (0.5 μM) was incubated with cellotetraose (0.5% wt/v) at different temperatures (20–70 °C) for 10 min in sodium phosphate buffer (pH 6.5). (**B**) Each cellobiohydrolase (0.5 μM) was incubated with cellotetraose (0.5% wt/v) at 37 °C in sodium citrate buffer (pH 4–6) or sodium phosphate buffer (pH 6.5–9) for 10 min. The reaction was stopped by heating at 99 °C for 10 min. The hydrolysis products were analyzed by HPAEC-PAD. The concentration of the released cellobiose was quantified by comparison of the peak area to a standard curve derived from known concentrations of cellobiose.

The end products of the cellobiohydrolases on cellotetraose suggested that they derive from a single catalytic round. Therefore, this substrate was used to determine the pH and temperature optima of each enzyme. Both Ra1589 and Ra1743 showed temperature optima ranging between 30–45 °C. Surprisingly, the temperature optimum of Ra3055 was at 55 °C, a temperature at which there was no activity for the other two proteins. Furthermore, we could measure relative activities of 20% and 10% at 60 °C and 65 °C, respectively, for Ra3055 (Fig. 2A). In the case of pH, the range was similar again for Ra1589 and Ra1743. The two enzymes showed broad activity range with the optima at 6.0–7.0. The pH optimum for Ra3055, in contrast was 6.5, with more than 80% activity being detected at pH 7.0 and 7.5. Furthermore, 40% of the maximum activity was detected at both pH 6.0 and pH 8.0 (Fig. 2B).

### Time course for the release of end products

**Figure 3:**
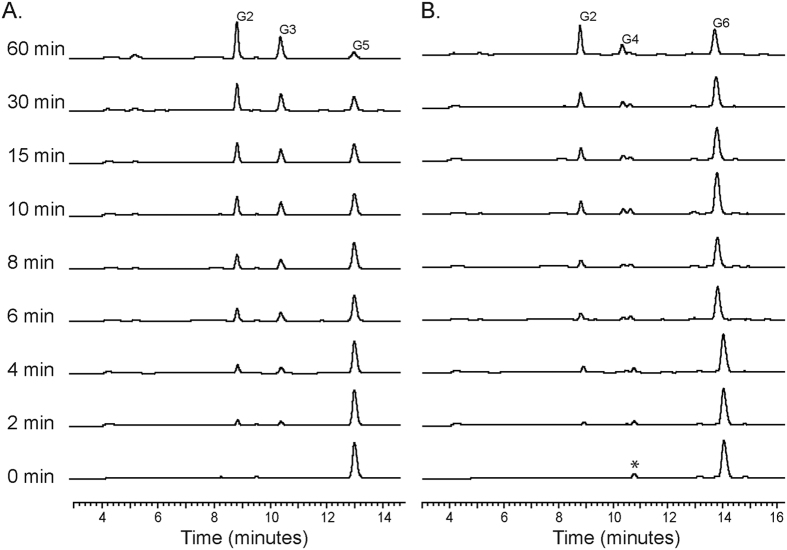
Time course analysis of cellopentaose and cellohexaose hydrolysis by Ra3055. The cellobiohydrolase (0.5 μM) was mixed with the cello-oligosaccharides (G5-G6) (0.5% wt/v) in sodium phosphate (pH 6.5). The reaction mixture was incubated at 37 °C for different incubation times (0–60 min) and the reaction was stopped by heating at 99 °C for 10 min. The end products of hydrolysis were analyzed by HPAEC-PAD. Products were identified by comparison of retention times to those of known cello-oligosaccharides. Asterisk denotes a contaminant present in the cellohexaose preparation.

We carried out an analysis to determine the time course for the release of end products for Ra3055, the most active of the three cellobiohydrolases, on an even number and odd number chain of oligosaccharides (Fig. 3). The *R. albus* 8 Ra3055 cleaved cellopentaose almost to completion within 60 minutes and over the period the end products were almost entirely cellobiose and cellotriose (Fig. 3A). On the other hand, the hydrolysis of cellohexaose was slow and the end products were more mixed (3 peaks) with most being cellobiose and cellotetraose (Fig. 3B).

### Truncational analysis to determine the contribution of various modules in Ra3055 to its enzymatic activity

**Figure 4:**
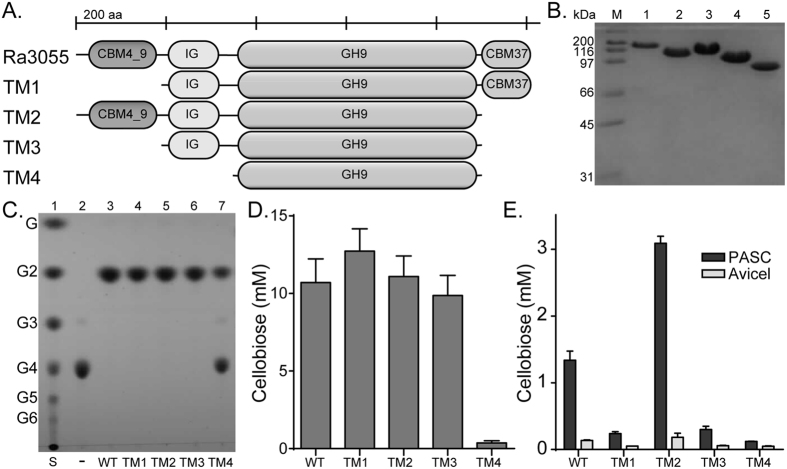
Truncational analysis of the cellobiohydrolase Ra3055. (**A**) Domain architectures of the truncational variants created from Ra3055. (**B**) SDS-PAGE of purified wild-type and truncational variants of Ra3055. Lane M, molecular mass marker; Lane 1, Ra3055 WT; Lane 2, Ra3055 TM1; Lane 3, Ra3055 TM2; Lane 4, Ra3055 TM3; Lane 5, Ra3055 TM4. (**C**) Thin layer chromatography for assessment of the hydrolytic activity of Ra3055 WT and its truncational variants against β-1,4 linked cellotetraose. Each enzyme (0.5 μM) was incubated with cellotetraose (0.5% wt/v) at 37 °C for 16 hours in sodium phosphate buffer (pH 6.5). The end products of hydrolysis and standards were resolved by thin layer chromatography. (**D**) Quantification of cellobiose released from cellotetraose (0.5% wt/v) after 10 minutes incubation with Ra3055 WT and truncational variants (0.5 μM) as analyzed by HPAEC-PAD. (**E**) Cellobiose released from Avicel and PASC by Ra3055 WT and its truncational variants. Each protein (0.5 μM) was incubated with the substrate (0.5% wt/v) in sodium phosphate buffer (pH 6.5) at 37 °C for 16 hours. The soluble fraction was separated by centrifugation and cellobiose was detected using HPAEC-PAD. The concentration of the released cellobiose was quantified by comparison of the peak area to a standard curve made with known concentrations of cellobiose.

A PCR approach was used to delete different modules in Ra3055 to determine their influence on the cellobiohydrolytic activity of the enzyme (Fig. 4A). All truncational variants (TM1, TM2, TM3, and TM4) were purified to near homogeneity based on SDS-PAGE analysis (Fig. 4B). Based on end point reactions and compared to the non-mutated enzyme (WT), removal of the N-terminal CBM4_9 (TM1) appeared to have no influence on the hydrolysis of cellotetraose. Similar observations were made with the deletion of the C-terminal CBM37 from the polypeptide (TM2). Furthermore, cleaving both the N-terminal CBM4_9 and the C-terminal CBM37 (TM3) and thus leaving only the Ig-domain and the GH9 catalytic module (TM3) also seem to have no impact on the catalytic activity of Ra3055. However, removal of all accessory domains (TM4) led to very little hydrolysis of cellotetraose (Fig. 4C,D). In contrast, analysis of the activities of the different truncational variants with polysaccharides as substrates yielded unexpected results. While all of the truncational mutants showed reduced activity on Avicel and PASC compared to the full length enzyme (WT), the TM2 mutant rather showed higher release of end products. In fact, the amount of cellobiose equivalents released by TM2 was about twice that of the full length protein, whereas with other variants there were several-fold decreases in hydrolysis of the polysaccharides (Fig. 4E).

### Enzyme kinetics parameters of the truncational variants

To gain better insights to the effect of the different deletions on the catalytic activity of Ra3055 as a cellobiohydrolase, the impact of the deletions in the polypeptide on the kinetic parameters of the enzyme were estimated with cellotetraose as the substrate. The removal of each of the CBMs led to an increase in the *k*_cat_ of the enzyme; however, in each case the *K*_m_ also increased with subsequent catalytic efficiency of TM1 being slightly higher while this parameter for TM2 was lower compared with the full length protein (Table 1). The TM3 mutant had a similar *K*_m_ as the full length polypeptide, and a slightly higher *k*_cat_ led to a similar catalytic efficiency as that of the full length polypeptide. Similar to the end point assay, cleaving of all the accessory modules, and thus leaving only the GH9 catalytic domain, led to a dramatic decrease in activity, with the catalytic efficiency dropping by close to 120-fold.

### Characterization of cellobiose phosphorylases from *R. albus* 8

**Figure 5:**
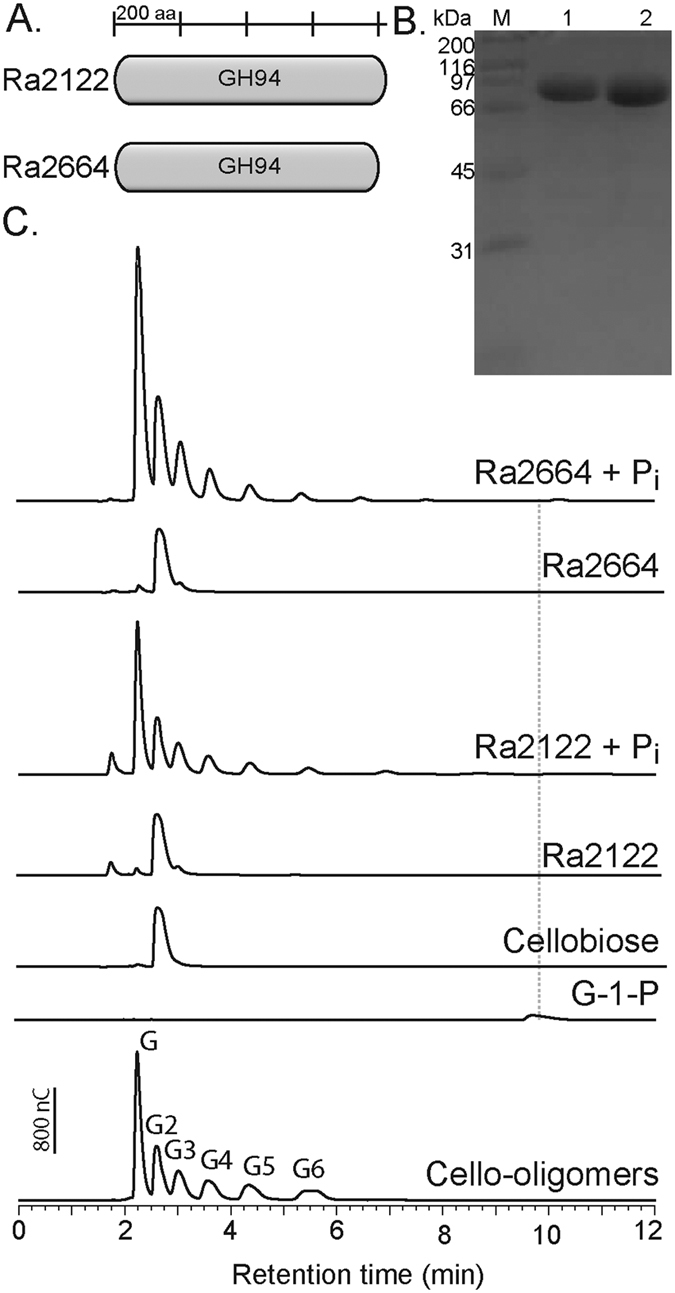
Cloning and characterization of two putative cellobiose phosphorylases from *Ruminococcus albus* 8. (**A**). Domain architecture of two putative cellobiose phosphorylases were predicted using Pfam (http://pfam.sanger.ac.uk). (**B**) SDS-PAGE of two putative cellobiose phosphorylases. Lane M, molecular mass marker; Lane 1, Ra2122; Lane 2, Ra2664. (**C**) Each protein (5 μM) was incubated with the substrate (10% wt/v) and 10 mM sodium phosphate in citrate buffer (pH 6.5 at 37 °C for 30 min. The products were analyzed using HPAEC-PAD. The end products were identified by comparing retention times to those of known cello-oligosaccharides and glucose 1-phosphate (grey line).

The GH9 enzymes, including Ra3055 with the highest catalytic activity, behaved as cellobiohydrolases with mainly cellobiose as end products. It was reasoned that *R. albus* 8 will encode enzymes that cleave the cellobiose to two glucose units for fermentation through the glycolytic pathway. A search of the genome of *R. albus* 8 yielded two genes encoding GH94 polypeptides (Fig. 5A) that are likely to function as cellobiose phosphorylases. The genes were expressed and the proteins were purified to near homogeneity (Fig. 5B). Each polypeptide was then investigated for the capacity to yield hydrolytic products from cellobiose. As shown in Fig. 5C, several peaks including glucose and other cello-oligosaccharides ranging from cellotriose to cellohexaose, were observed with the incubation of Ra2122 and Ra2664 on cellobiose in the presence of inorganic phosphate. While a clear peak representing glucose-1-phosphate (G-1-P) was not observed for Ra2122, a discernible peak for G-1-P was present from the end products of Ra2664 on cellobiose.

### Hydrolysis of a cellulosic substrate with an endoglucanase, a cellobiohydrolase and cellobiose phosphorylases from *R. albus* 8

**Figure 6:**
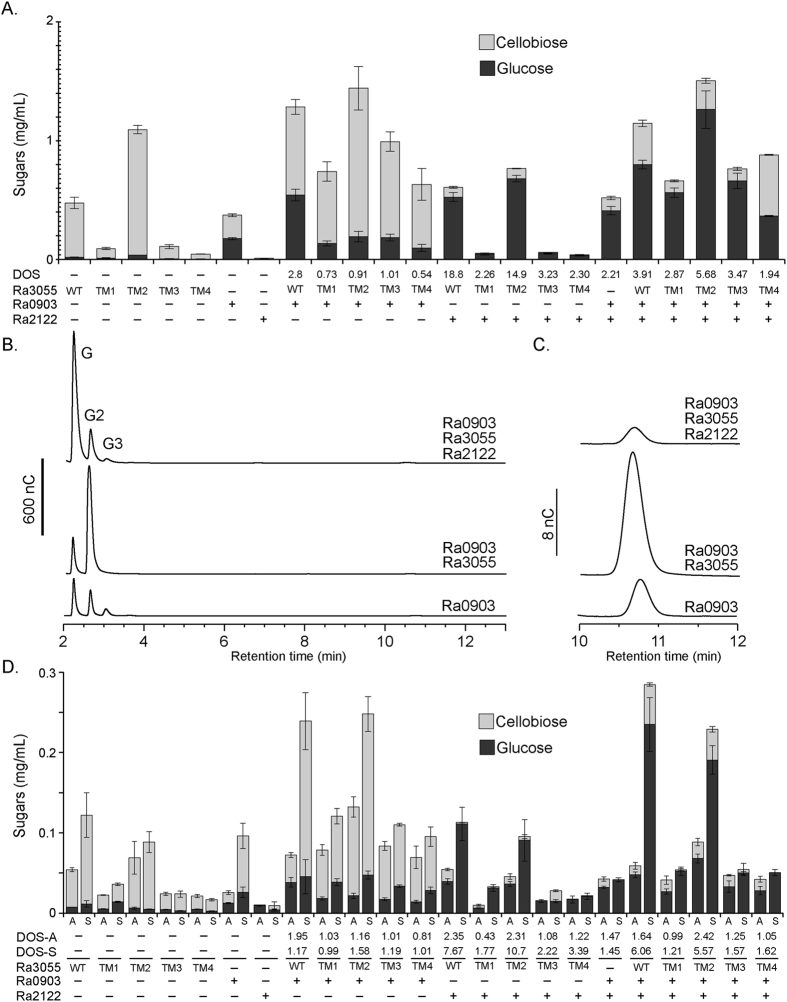
Synergistic activity of Ra3055, Ra0903, and Ra2122 on cellulosic polysaccharides. (**A**) Combinations of Ra3055, Ra0903, and Ra2122 (0.5 μM each) were incubated with PASC (0.5% w/v) in sodium phosphate buffer (pH 6.5) at 37 °C for 16 hours. The soluble fraction was analyzed by HPAEC-PAD. The end products of hydrolysis were identified by comparing their retention time to the retention times of known oligosaccharides. Degree of synergy was calculated for the release of glucose (DOS = [glucose released by enzyme mixture]/Σ [concentration of glucose released from incubation with the individual enzymes in the mixture]). (**B**) All combination of Ra3055, Ra0903, and Ra2122 (0.5 μM each) were incubated with PASC (0.5% w/v) in sodium phosphate buffer (pH 6.5) at 37 °C for 16 hours. (**C**) All combination of Ra3055, Ra0903, and Ra2122 (0.5 μM each) were incubated with Avicel (**A**) and pretreated sugarcane bagasse (S) (0.5% w/v) in sodium phosphate buffer (pH 6.5) at 37 °C for 16 hours. The concentration of the released sugars was quantified by comparison of the peak area to a standard curve derived using known concentrations of glucose and cellobiose. Degree of synergy on Avicel (DOS-A) and sugarcane bagasse (DOS-S) was calculated for the release of glucose (DOS = [glucose released by enzyme mixture]/Σ [concentration of glucose released from incubation with the individual enzymes in the mixture]).

The endoglucanases, cellobiohydrolases, and cellobiose-phosphorylases are likely to cooperate to release fermentable sugars for utilization by *R. albus* 8. Thus, the synergistic activity of the most active enzymes of each enzyme family (endoglucanase (Ra0903), cellobiohydrolase (Ra3055) and cellobiose phosphorylase (Ra2122)) was tested. Furthermore, the different truncational variants of the cellobiohydrolase Ra3055 were tested for their effects on the synergy of the three enzymes (Fig. 6A). The Ra3055 enzyme (designated WT) released mostly cellobiose from the PASC. Furthermore, the truncational variant TM2 of Ra3055 released about twice the amount of cellobiose released by the full length enzyme or WT. A combination of the cellobiohydrolase and its truncational variants (TM1, TM2, TM3, and TM4) with the endoglucanase resulted in large increases in both cellobiose and glucose (Fig. 6A). In contrast, a combination of the truncational variants and the cellobiose-phosphorylase (0.5 μM each) led to release of mostly glucose, i.e., >90% glucose present in the reaction mixture. A combination of the 3 enzymes led to release of higher amounts of glucose in the reaction mixture. It is also of interest to note that in each case of the combinations of enzymes on PASC, the presence of TM2 resulted in the highest amounts of end products.

In Fig. 6B, it is very clear from the chromatogram that the mixture of the endoglucanase and the cellobiohydrolase (Ra0903 and Ra3055) led to a large increase in cellobiose production. Combining the two enzymes with the cellobiose phosphorylase then led to most of the cellobiose being converted to glucose. We increased the sensitivity around the retention time at which glucose-1-phosphate elutes (Fig. 6C) to determine the presence of glucose-1-phosphate. However, we observed a peak that appears to be present at the elution time of G-1-P even in the absence of the cellobiose phosphorylase in the reaction mixture. Thus, the release of this end product in the three-enzyme reaction mixture was inconclusive.

The synergistic activity of the 3 enzymes was further assessed with the crystalline cellulose Avicel and also pretreated sugarcane bagasse as substrates (Fig. 6C). Similar to the experiment with PASC, the endoglucanases by themselves and also in combination with the cellobiohydrolase and its truncational variants led to mostly the release of cellobiose, whereas the incubation with a mixture of the cellobiohydrolase and its truncational derivatives with the cellobiose-phosphorylase resulted in mostly glucose (Fig. 6C). As expected, the reaction with all three enzymes resulted in the highest release of end products and these were mostly glucose. As observed before, the WT and TM2 variant always yielded higher end products than all truncational variants when present in a reaction mixture. In Avicel and pretreated sugarcane bagasse, however, the mixture with the WT enzyme appears to yield more end products than the reaction with TM2 (Fig. 6D), suggesting importance of the CBM4_9 in hydrolysis of complex substrates. We calculated the degree of synergy (DOS) based on release of glucose as the final end product. As expected, the presence of the cellobiose phosphorylase in the enzyme mixture led to a very high degree of synergy compare to other enzyme mixtures lacking the cellobiose phosphorylase or Ra2122. This observation clearly demonstrated the function of the cellobiose phosphorylase in converting most of the end products of the endoglucanase and the cellobiohydrolase to the fermentable sugar glucose. It is also noteworthy that the degree of synergy in the presence of the cellobiose phosphorylase was generally higher on the pretreated sugar cane bagasse than on the crystalline cellulose Avicel.

### Substrate binding activity of Ra3055 and its truncational variants

**Figure 7:**
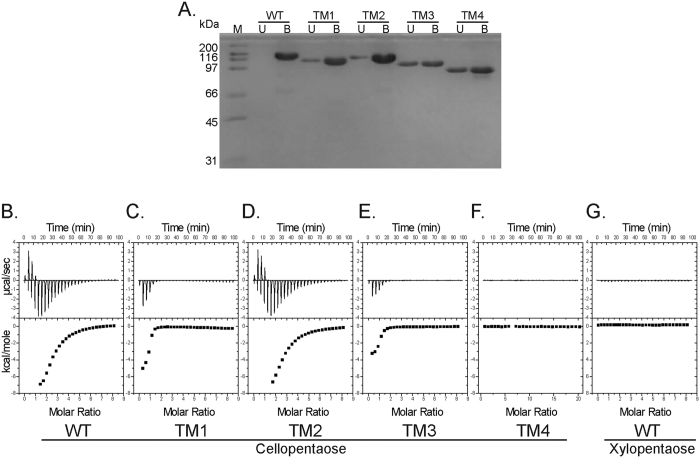
Binding of wild-type and truncational variants of the cellobiohydrolase Ra3055 to polysaccharides and oligosaccharide. (**A**) Binding of Ra3055 WT and its truncational variants to Avicel. Each protein (1 mg/ml) was incubated with Avicel (10% wt/v) at 4 °C for 1 hour. The mixture was centrifuged and the supernatant containing the unbound fraction (U) and the pellet containing the bound fraction (**B**) were analyzed by SDS PAGE. (**B**–**G**) Isothermal titration calorimetry was employed to determine the binding of Ra3055 WT and truncational variants to cellopentaose and the binding of Ra3055 WT to xylopentaose. The substrate (2 mM) was injected in 10 μl increments into the sample cell containing purified proteins (50 μM) in a stepwise fashion, and the sequential peaks corresponding to each injection were recorded.

Since the cellobiohydrolases have similar modular architecture, the truncational mutants were investigated to determine the contributions of the different modules to substrate binding. The first experiment (SI Fig. 1) involved migration of the proteins of interest through substrate infused non-denaturing PAGE. Here the results were inconclusive, since the electrophoresis with the substrate infused and non-infused gels yielded the same migration pattern. The proteins were, therefore, investigated for their capacity to bind to the crystalline cellulose Avicel. On reacting with Avicel, the full length protein (WT), and the truncational derivatives TM1 and TM2 clearly bound to the Avicel with very little amount of the polypeptide remaining in solution (Fig. 7A). Although with TM3 and TM4 there was binding to substrates, increased amounts of the proteins were also found in solution (U under TM3 and TM4). Further binding analysis was carried out through isothermal titration calorimetry (ITC) with an oligosaccharide, cellopentaose, used as the ligand (Fig. 7B–G). Here also the WT, TM1 and TM2 showed binding to the substrate and a weaker binding was observed with TM3, while for TM4 no binding was detected (SI Table 2). The capacity of the full length protein to bind to xylopentaose was determined, and as shown in Fig. 7G, the polypeptide failed to bind this substrate.

### Expression of cellobiohydrolases and cellobiose phosphorylases in *R. albus* 8

**Figure 8:**
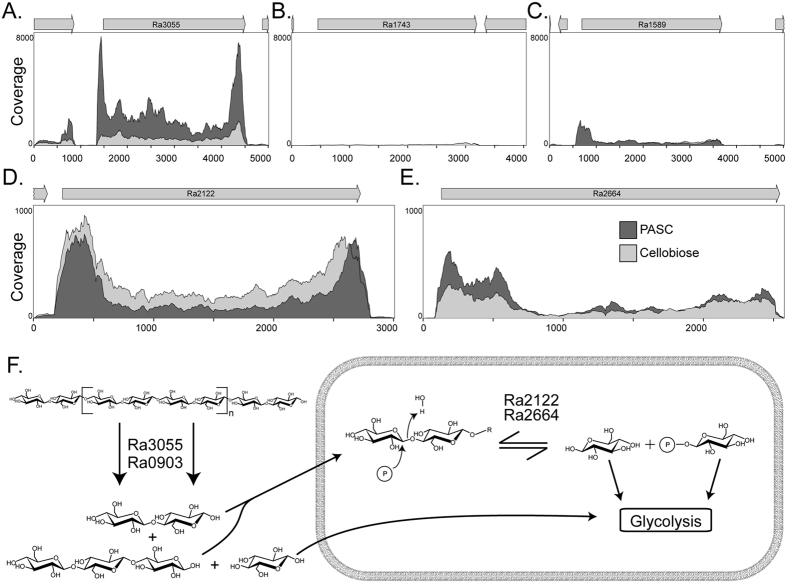
Expression analysis of the cellobiohydrolases and cellobiose phosphorylases of *R. albus* 8 grown on PASC and cellobiose. RNA-Seq was performed on *R. albus* 8 cells grown on PASC or cellobiose as the sole carbon source. The resulting coverage maps for the cellobiohydrolases, Ra3055 (**A**), Ra1743 (**B**), and Ra1589 (**C**), as well as the cellobiose phosphorylases Ra2122 (**D**) and Ra2664 (**E**) were displayed. The RNA was extracted from *R. albus* 8 during the logarithmic phase and sequenced at the W. M. Keck Center for Comparative and Functional Genomics. Analysis of RNA-seq data was performed using CLC genomics workbench. (**F**) Schematic diagram for cellulose utilization by *R. albus* 8.

The expression of the cellobiohydrolases and cellobiose-phosphorylases in *R. albus* 8 during growth on cellobiose and phosphoric acid swollen cellulose (PASC) were examined through RNAseq analysis. Of the three cellobiohydrolases, only Ra3055 was clearly expressed on both substrates (Fig. 8A). Some expression was observed with Ra1589 on PASC; however, the expression was very low compared with Ra3055 (Fig. 8C). In contrast, expression of Ra1743 was not detected under both culture conditions (Fig. 8B). RNA transcripts of both cellobiose phosphorylases (Ra2122 and Ra2664) were, however, detectable during growth on either PASC or cellobiose, demonstrating the importance of these downstream enzymes during fermentation of both substrates.

## Discussion

In the rumen, *Ruminococcus albus* 8, a fibrolytic organism is one of the most important plant cell wall degrading bacteria. Although the ability of this bacterium to degrade plant cell wall polysaccharides is well known, the mechanism of cellulose degradation by *R. albus* 8 is little understood. Through analysis of the draft genome sequence of *R. albus* 8, we previously uncovered many genes predicted to be involved in cellulose depolymerization, including 17 putative endoglucanases, three putative cellobiohydrolases, and two putative cellobiose phosphorylases. The endoglucanases of *R. albus* 8, which represent the enzymes that initially attack the cellulose backbone were recently characterized^28^. To gain insights into the role of accessory enzymes that likely function in synergy with the endoglucanases to depolymerize cellulose, we cloned and heterologously expressed the genes encoding the three putative cellobiohydrolases and two putative cellobiose phosphorylases for biochemical analysis of the gene products.

The cellobiohydrolases encoded by *R. albus* 8 belong to GH9, for which members display a broad spectrum of substrate specificity. The enzymatic activities of the GH9 further differs based on fusion of other modules, such as carbohydrate-binding modules (CBM), in the same polypeptide^29^. The catalytic modules contain an (α/α)_6_ barrel structure and other non-catalytic domains may be appended N- or C-terminally to the GH9 domain. The putative cellobiohydrolases (Ra3055, Ra1589 and Ra1743) studied here have an N-terminal CBM4_9 domain followed by an Ig-like domain, the GH9 catalytic module and a CBM37 at the C-terminus^27^. Note, however, that the last module, i.e., the CBM37 is missing in Ra1743. The structure of the *R. albus* 8 enzymes are similar to those found in other Firmicutes, including the Cel9E and Cel9V of *Clostridium cellulolyticum*, except for the presence of dockerin domains in place of the CBM37^30^. The swapping of the CBM37 for dockerins is a unique feature of *R. albus* species which utilize the CBM37 for direct attachment to the bacterial cell wall^27^, while dockerins are indirectly attached to a bacterial cell via cellulosomes. During hydrolysis of substrates, Ra3055, Ra1589, and Ra1743 exhibited far higher activities on amorphous cellulose (PASC) compared to crystalline cellulose (Avicel) and released cellobiose as a major end product (Fig. 1D). The activites of the *R. albus* 8 proteins are analogous to the *C. cellulolyticum* proteins, i.e., they release predominantly cellobiose from cellulosic polysaccharides^30^. Other GH9 cellulases with similar domain architectures, including CelK and CenC from *Clostridium thermocellum*, and CelE from *Clostridium cellulolyticum* have been reported to release cellobiose from cellulosic substrates^11^,^13^. As previously reported in multi-domain cellulases, the GH9 enzymes of *R. albus* 8 likely act first by a random cleavage of cellulose and then employ a processive mode of action to release cellobiose^11^,^13^,^31^,^32^. In contrast to another well known ruminal cellulolytic bacterium, the Cel9D of *Fibrobacter succinogenes* S85 exhibits a processive release of glucose from the reducing ends of cello-oligosaccharides and cellulose^33^,^34^.

The GH9 enzymes are commonly appended with non-catalytic modules including one or more CBMs and Ig-like modules. The CBM enhances the efficiency of the enzyme by increasing both the proximity of the enzyme to the substrate and the enzyme concentration on the surface of an insoluble substrate^31^,^35^,^36^. The Ig-like module impacts the catalytic activity of cellulases by modifying the structural stability of the enzymes^37^. In the present study, truncational analysis of Ra3055, the most highly expressed cellobiohydrolase relative to other glycoside hydrolases, was employed to identify the role of individual domains to catalysis and binding of amorphous cellulose and soluble cello-oligosaccharides. A series of truncational mutants of Ra3055, lacking individual or multiple domains were made (Fig. 4). The removal of the CBM4_9, i.e., in Ra3055 TM1 and TM3, resulted in a drastic decrease in hydrolysis of amorphous and crystalline cellulose, while no decrease was observed in cellotetraose degradation. This finding suggests that this CBM is essential for the recognition and degradation of the polymeric cellulose. Previous reports on truncational variants in cellobiohydrolases of *Clostridium thermocellum* (CbhA) and *Clostridium cellulovorans* (EngK and EngM) and *C. cellulolyticum* (CelE) observed similar finding, i.e., a decrease in activity or complete loss of activity on cellulosic substrates^11^,^20^,^32^,^38^. The crystal structure of a CBM4 fused to the Ig-like domain from the CbhA of *C. thermocellum* revealed that CBM4 efficiently binds to amorphous regions of cellulose^39^. Contrasting these results, the truncational variant lacking CBM37, i.e., Ra3055 TM2, had an increased activity on swollen cellulose (Figs 4 and 6). Activity on Avicel and sugarcane bagasse had a similar phenomenon, with Ra3055 TM2 exhibiting similar activity to WT, while Ra3055 TM1 and TM3 only possessed very low activities compared with WT (Fig. 6). This observation may be the result of the promiscuous nature of CBM37 in binding to a large number of polysaccharides^40^. This property of CBM37 may titrate Ra3055 away from the preferred amorphous regions of hydrolysis, to which CBM4_9 has high affinity^39^. Thus, the loss of CBM37 increases the affinity to the preferred binding regions of CBM4_9 (amorphous) and therefore leading to more robust hydrolysis. Alternatively, the CBM37 domain is implicated as a shuttle used by the organism to transfer glycoside hydrolases to the plant cell wall when in close proximity, as evidenced by the CBM37 for the peptidoglycan of *R. albus* 8^27^. Here, the CBM37 may adhere the enzyme to the peptidoglycan and only hydrolyze amorphous cellulose in direct contact with the bacterium, thus decreasing competition by other organisms in its community for the end products of hydrolysis. In terms of the physiology of *R. albus* 8, this suggests that if the CBM37 is removed, the enzyme may be more versatile; however, this may also decrease fitness of the bacterium by reducing its effectiveness in capturing energy from its environments. The truncational variant lacking all accessory domains, Ra3055 TM4, had very low activity on all tested substrates, reflecting the importance of the appended modules to the overall function of the polypeptide. This phenomenon was also seen in truncational and site-directed mutants of the CbhA of *C. thermocellum* and also further revealed that hydrophilic and hydrophobic interactions between the Ig-like module and the catalytic module are required for enzymatic activity^37^,^41^.

Due to the complexity of the ruminal microbial community, an efficent system for nutrient acquisition and utilization is essential. To conserve energy during the fermentation of cellulose, *R. albus* degrades cellobiose through phosphorolysis as well as hydrolysis. Previous reports predicted that almost 75% of consumed cello-oligomers were degraded through phosphorolytic cleavage^42^. The conversion of cellobiose to glucose and glucose-1-phosphate (G-1P) is catalyzed by cellobiose phosphorylase (CBP) and cellodextrin phosphorylase, previously characterized from ruminal and soil microbes^6^,^7^,^43^. From the draft genome sequence of *R. albus* 8, we identified two genes encoding putative cellobiose phosphorylases (Ra2122 and Ra2664). The amino acid sequences of the polypeptides were almost identitcal (96% identity) to the cellobiose phosphorylases (Rumal_2403 and Rumal_0187) of *R. albus* 7 and belong to GH94. Furthermore, homologs of these proteins have been characterized from *R. albus* NE1^7^. The *R. albus* 8 cellobiose phosphorylases showed similar activity on cellobiose, releasing glucose and a range of cello-oligosaccharides through transglycosylation reactions (Fig. 5). A similar activity was observed in the purified cellodextrin phosphorylase from *R. albus* NE1 and the cell extract of *R. albus* B199 when the proteins were incubated with cellobiose and glucose-1-phosphate. In both cases, a decrease in cellobiose with a concomitant increase in cellotriose, cellotetraose, and cellopentaose was observed^42^,^44^. Differences in substrate recognition between Ra2122 and Ra2664 were apparent when we analyzed the hydrolysis patterns on longer chain oligosaccharides, i.e., cellotriose and cellotetraose (SI Fig. 2). Although transglycosylation products were observed for both enzymes, differences in activity were visible when longer chain oligosaccharides were used as substrates. For example, Ra2664 released predominantly glucose from cellotriose and cellotetraose while Ra2122 produced small amounts of longer chain oligosaccharides, indicating Ra2664 as a cellodextrin phosphorylase. Since Ra2122 was only able to efficiently degrade cellobiose, this enzyme may act primarily as a cellobiose phosphorylase within the organism, in agreement with previous reports^6^,^7^,^43^,^44^.

To assess synergistic effects, combinatorial mixtures containing an endoglucanase (Ra0903), a cellobiohydrolase (Ra3055), and the cellobiose phosphorylase (Ra2122) were co-incubated with cellulosic substrates (Fig. 6A,D). With Ra0903 and Ra3055, synergistic release of cellobiose and glucose was observed when compared to the individual enzymes, and addition of the cellobiose phosphorylase increased the proportion of glucose in the final products. We predict that the cellobiose released as the predominant end product by Ra0903 and Ra3055 is transported into the cytoplasm by cellobiose transporters for subsequent phosphorolysis by Ra2122 and Ra2664 to glucose and G-1-P for metabolism through glycolysis^42^. A functionally homologous system was recently characterized from an uncultured member of the ruminal Bacteroidetes derived from metagenomic sequencing. Unlike Ruminococci, *Bacteroides* usually possess genetic loci encoding the genes necessary for the complete deconstruction of specific polysaccharides in a discrete PUL. One such locus (from the metagenomic data) is implicated in the degradation of cellulose and contains an endoglucanase (GH5 catalytic domain only), a GH9 cellobiohydrolase (Ig-like domain appended to a GH9 catalytic domain) and a cellobiose phosphorylase (GH94), with a synergistic activity described for the endoglucanase and the cellobiohydrolase^45^.

In this report, we provide biochemical evidence for the enzymes involved in cellulose depolymerization by a prominent ruminal bacterium *R. albus* 8. Through truncational analysis, we gained insight into the contribution of each domain of the highly expressed cellobiohydrolase Ra3055 towards cellulose deconstruction. Examination of hydrolytic synergy, through enzyme mixtures, showed how cellulose can be efficiently converted into fermentable sugars by this bacterium. Thus, our comprehensive biochemical analysis sheds light on the enzymatic strategy employed by *R. albus* 8 for efficient energy capture from cellulose.

## Materials and Methods

### Materials

*R. albus* 8 genomic DNA was obtained from the Department of Animal Sciences, University of Illinois at Urbana-Champaign (20). *Escherichia coli* DH5α and *E. coli* BL21-CodonPlus(DE3) RIPL competent cells and PicoMaxx high-fidelity DNA polymerase were purchased from Stratagene (La Jolla, CA). The pET-46 Ek/LIC vector kit was obtained from Novagen (San Diego, CA). The QIAprep Spin Miniprep kit was obtained from Qiagen (Valencia, CA). The RNAprotect^®^ bacterial reagent and RNeasy mini kit were from Qiagen (Valencia, CA), the MicrobExpress kit was from LifeTechnologies (Carlsbad, CA), and the Isopropyl β-D-1-thiogalactopyranoside (IPTG) was purchased from Gold Biotechnology (St. Louis, MO). The 1,4-β-cello-oligosaccharides, xylopentaose and wheat arabinoxylan (WAX) were purchased from Megazyme (Bray, Ireland). The Amicon Ultra-15 centrifugal filter units with 30-kDa- and 50-kDa-molecular-mass cutoffs (MMCOs) were obtained from Millipore (Billerica, MA). The Avicel PH-100 was purchased from Sigma-Aldrich (St. Louis, MO). All other reagents were of the highest possible purity and were purchased from Fisher Scientific (Pittsburgh, PA).

### Gene cloning

The putative cellobiohydrolases (Ra3055, Ra1589 and Ra1743) and cellobiose phosphorylases (Ra2122 and Ra2664) were cloned as previously described^46^. Briefly, genes of interest were amplified with primers listed in supplementary table1, using PicoMaxx high fidelity PCR kit (Stratagene) and cloned into pET-46b vector (Novagen) as described by the manufacturer’s protocol. Resulting colonies were isolated on Lysogeny broth (LB) agar plates containing ampicillin (100 μg/ml) and inoculated into LB liquid medium containing ampicillin (100 μg/ml) and grown at 37 °C for 16 hours. Cells were collected by centrifugation (3220 × g, 15 min, 4 °C) and plasmids were extracted using the QIAprep Spin Miniprep kit (Qiagen, Valencia, CA). Plasmids were sequenced at the W. M. Keck Center for Comparative and Functional Genomics at the University of Illinois at Urbana-Champaign to confirm presence of the correct gene sequence.

### Gene expression and protein purification

Proteins were expressed as previously described^23^. Briefly, plasmids were freshly transformed into *E. coli* BL-21 CodonPlus (DE3) RIPL cells using the heat shock method. Cells were grown on LB agar plate with ampicillin (100 μg/ml) and chloramphenicol (50 μg/ml) and 5 colonies were inoculated into 10 mL of LB broth containing the same antibiotics at the same concentrations. After 6 hours of growth at 37 °C with shaking, 1 L of LB broth was inoculated with the 10 ml pre-culture and grown (at 37 °C with shaking) until the optical density at 600 nm (OD_600_) reached 0.3–0.5, when the cells were induced with 0.1 mM of IPTG. Upon induction, the culturing of the cells was shifted to 16 °C for 16 hours. Then, the cells were pelleted by centrifugation (4,000 × g, 30 min, 4 °C) and re-suspended in 30 mL of binding buffer (20 mM Tris-HCl, 300 mM NaCl, pH 7.9). For the cellobiose phosphorylases, the cell pellets were re-suspended in 20 mM MES buffer containing 300 mM NaCl (pH 7.9). The cell suspensions were subjected to two passages through an EmulsiFlex C-3 cell homogenizer (Avestin, Ottawa, Canada), and the cell lysate was clarified by centrifugation at 20,000 × g for 30 min at 4 °C.

The recombinant cellobiohydrolases (Ra3055, Ra1589 and Ra1743) were then purified using Ni-affinity column (Histrap FF 5 ml, GE Healthcare, Piscataway, NJ) attached to an AKTAxpress FPLC (GE Healthcare, Piscataway, NJ). The proteins that bound to the column were eluted using an elution buffer composed of 20 mM Tris-HCl, pH 7.9, 300 mM NaCl and 500 mM imidazole. The partially purified proteins were subjected to anion-exchange chromatography using a 5-ml HiTrap Q HP column (GE Healthcare, Piscataway, NJ) with a binding buffer of 20 mM Tris-HCl, pH 7.9 and an elution buffer composed of the binding buffer supplemented with NaCl at 1 M. Finally, the eluted proteins were loaded onto a HiLoad 16/60 Superdex 200 gel filtration column (GE Healthcare, Piscataway, NJ), and the eluted proteins were stored in the gel filtration buffer composed of 20 mM Tris-HCl, 150 mM NaCl, pH 7.5. To purify the recombinant cellobiose phosphorylases (Ra2122 and Ra2664), the cell lysate was subjected to Ni-affinity column (Histrap FF 5 ml, GE Healthcare, Piscataway, NJ) as described above with a modified binding buffer (20 mM MES-NaOH, pH 7.9, 300 mM NaCl) and an elution buffer composed of the binding buffer supplemented with 500 mM imidazole. The purified fractions were pooled, concentrated and subjected to gel filtration chromatography (HiLoad 16/60 Superdex 200 column) with a buffer composed of 20 mM MES-NaOH, 150 mM NaCl (pH 7.5). Purified proteins were stored in the gel filtration buffer at 4 °C until used for enzymatic reactions. The putative endoglucanase (Ra0903) was purified by a two-step chromatographic method as previously described^28^. The purity of enzymes was analyzed by sodium dodecyl sulfate-polyacrylamide gel electrophoresis (SDS-PAGE), and the protein bands were visualized by staining with Coomassie brilliant blue G-250. The protein concentrations were calculated based on the molecular mass and computed extinction coefficients as described in our previous reports. The extinction coefficients for Ra0903, Ra3055, Ra1589, Ra1743, Ra2122 and Ra2664 were 202,515 M^−1^cm^−1^, 251,460 M^−1^cm^−1^, 239,110 M^−1^cm^−1^, 98,537 M^−1^cm^−1^, 161,175 M^−1^cm^−1^ and 155,800 M^−1^cm^−1^ respectively.

### Preparation of phosphoric acid swollen cellulose (PASC)

Phosphoric-acid swollen cellulose (PASC) was prepared from Avicel (Fluka,PH-101) according to a previously published method^47^. Avicel (10 g) was dissolved in 250 mL of phosphoric acid. After stirring for 1 hr at 4 °C, the solution was diluted with 3.75 L of cold water. After stirring for 1 hr at 4 °C, the swollen cellulose was collected by centrifuging the mixture at 1,000 RPM for 20 minutes. The amorphous cellulose was washed 3 times with ultrapure water. The resultant slurry was neutralized with 1% NaHCO_3_ and then washed with ultrapure water. The slurry was re-suspended in 50 mM phosphate buffer (pH 6.5) containing 0.01% sodium azide and stored at 4 °C.

### Evaluation of hydrolysis of cello-oligosaccharides

To evaluate the hydrolytic activity of putative cellobiohydrolases against the cello-oligosaccharides (G2–G6), each cello-oligosaccharides (G2–G6) at a final concentration of 5 mg/ml was dissolved in phosphate buffer (pH 6.5, 50 mM, NaCl, 150 mM) and reactions were initiated by the addition of enzyme (0.5 μM final concentration) and the mixtures incubated at 37 °C for 16 hr. A negative control lacking enzyme was prepared for each cello-oligosaccharide reaction mixture. Enzymatic reactions were stopped by heating at 99 °C for 10 min and 1-μl aliquots of samples were spotted onto silica gel thin-layer chromatography (TLC) plates (60 Å, 250-μm thickness) (Whatman, Piscataway, NJ). The end products of the hydrolysis as well as oligo-saccharide (G1–G6) standards were resolved with a mobile phase composed of n-butanol, acetic acid, and water (3:2:1). The plates were then dried, and products were visualized by spraying with a mixture of sulfuric acid (10%, v/v), orcinol (0.1%, w/v), and methanol (50%, v/v) and developed by heating at 80 °C for 10 min.

To evaluate the hydrolytic patterns of the cellobiohydrolase (Ra3055) over time, the cellobiohydrolase (0.5 μM) was mixed with the cello-oligosaccharides (G5–G6) at a final concentration of 0.5% w/v in sodium phosphate (pH 6.5). The reaction mixture was incubated at 37 °C for different incubation times (0–60 min) and the reaction was stopped by heating at 99 °C for 10 min. The products of hydrolysis were first diluted 100-fold and then analyzed using high-performance anion exchange chromatography (HPAEC) (Beckman Coulter, Fullerton, CA) equipped with a CarboPac PA1 guard column (4 by 50 mm) and a CarboPac PA1 analytical column (4 by 250 mm) from Dionex Corporation (Sunnyvale, CA) and with a pulsed amperometric detector (PAD) (ESA Biosciences, Chelmsford, MA). Peak retention times and peak areas of samples were compared with standard oligosaccharides.

### Evaluation of hydrolysis of cellulose polysaccharides

To evaluate the hydrolytic activity of putative cellobiohydrolases against the natural cellulose polysaccharides. Each polysaccharide (Avicel and PASC) at final concentration of 0.5% w/v were dissolved in 50 mM phosphate buffer (pH 6.5, NaCl, 150 mM). Reactions were initiated by the addition of enzyme (0.5 μM final concentrations) and the mixtures were incubated at 37 °C for 16 hr. The reaction mixtures were then heat inactivated at 99 °C for 10 min and centrifuged for 10 min at 15,000 × *g*. The hydrolytic products in the supernatant were analyzed using high performance anion exchange chromatography (HPAEC) equipped with pulsed amperometric detector (PAD) as described above. The concentration of the released cellobiose was quantified by comparison of the peak area to a standard curve derived from known concentrations of cellobiose.

### Determination of optimal pH and temperature

The buffers used to study the pH profiling of the three putative cellobiohydrolases were as follows: 50 mM sodium citrate, 150 mM NaCl (pH 4.0–6.0), 50 mM sodium phosphate, and 150 mM NaCl (pH 6.5–9.0). Each enzyme at a final concentration of 0.5 μM was incubated with cellotetraose (0.5% w/v) at 37 °C for 10 min. The reaction was stopped by heating at 99 °C for 10 min. The hydrolytic products were analyzed by HPAEC-PAD. The concentration of the released cellobiose was quantified by comparison of the peak area to a standard curve derived with known concentrations of cellobiose, and the initial activities were calculated. The optimal temperatures were determined by incubating each enzyme at a final concentration of 0.5 μM with cellotetraose (0.5% w/v) in a buffer of the optimal pH at temperatures ranging from 20 to 70 °C with an interval of 5 °C.

### Truncational analysis of the cellobiohydrolase Ra3055

To evaluate the binding affinity and function of carbohydrate binding modules in the cellobiohydrolase Ra3055, truncational variants were created and tested for both catalytic and binding activity. The nucleotide sequences encoding the truncated variants of Ra3055 were amplified by PCR with the primers listed on supplementary table 1. The PCR amplicon was cloned into the pET46 vector using ligation independent cloning as described above. The recombinant proteins from each cell lysate containing the truncated variant was purified by a three step chromatography procedure as described above, and the purity was assessed by SDS/PAGE. The protein concentrations were calculated based on the molecular mass and computed extinction coefficients. The extinction coefficients for TM1, TM2, TM3 and TM4 were 198,270 M^−1^cm^−1^, 226,020 M^−1^cm^−1^, 172,830 M^−1^cm^−1^ and 158,390 M^−1^cm^−1^ respectively. The enzymatic activities of each truncational variant on cellotetraose and on cellulose (PASC and Avicel) were determined as described above.

### Binding affinity of the wild-type and truncational variants of the cellobiohydrolase Ra3055 on soluble polysaccharides

To determine the binding of the wild-type and truncational variants of the cellobiohydrolase Ra3055 to soluble polysaccharides, non-denaturing polyacrylamide gel electrophoresis was used. Polyacrylamide gels (6%) were infused with carboxymethyl cellulose (CMC) or wheat arabinoxylan (WAX) at a concentration of 0.25% w/v. Each polypeptide and bovine serum albumin (2 μg) were loaded into wells and electrophoresed for 2 hours at 100 V at 4 °C. The gels were stained with Coomassie brilliant blue G-250 to visualize the protein bands. The migration patterns of enzymes were compared between substrate-infused and non-substrate infused polyacrylamide gels using BSA as standard.

### Binding affinity of wild-type and truncational variants of putative cellobiohydrolase Ra3055 on insoluble polysaccharide (Avicel)

Insoluble Avicel was prepared as follows: briefly, one gram of substrate was stirred in 100 ml of distilled water for 12 h. After centrifugation at 4,000 × g for 10 min, the precipitate was further washed with 100 ml of distilled water and centrifuged (4,000 × g, 10 min). The supernatant was decanted. The pellet containing the insoluble fractions were lyophilized for 24 hr and then ground into a fine powder in a mortar, producing insoluble Avicel. The binding of proteins to insoluble substrate was carried out as follows, each enzyme (2 μg/ml) was incubated with Avicel (2% w/v) for 1 hour at 4 °C. After the incubation, each reaction mixture was centrifuged at 25,000 × g for 10 minutes to pellet the substrate with bound proteins. The residual proteins present in the supernatant represented the unbound fraction of loaded proteins. Weakly bound enzymes in the pellet were removed by washing with reaction buffer (50 mM sodium phosphate, 150 mM NaCl, pH 6.5). The polypeptides associated with the substrate (in pellet) were used as the bound fraction and were eluted and compared to the unbound fraction via SDS-PAGE.

### Isothermal titration calorimetric analysis of wild-type and truncational variants of the putative cellobiohydrolase Ra3055

Isothermal titration calorimetric (ITC) analysis was performed using a VP-ITC microcalorimeter with a 1.4 mL cell volume from MicroCal, Inc. The proteins were dialyzed with phosphate buffer (50 mM sodium phosphate, 150 mM NaCl, pH 7.5) and the oligosaccharides (cellopentaose and xylopentaose) were dissolved in the same buffer. The proteins (50 μM) were then injected with 28 successive 10 μL aliquots of ligand (2 mM) at 300-s intervals. The data were fitted to a nonlinear regression model using a single binding site (MicroCal Origin software). The thermodynamic parameters were calculated using the Gibbs free energy equation (ΔG = ΔH−TΔS), and the relationship ΔG = −RTln(K_a_).

### Kinetic analysis of wild-type and truncational variants of the cellobiohydrolase Ra3055

To determine the kinetic parameters of the wild-type Ra3055 and its truncational variants, each enzyme (0.5 μM) was incubated with varying concentration of cellotetraose (0.01 to 30 mM) for 10 min. The velocity of each reaction was determined by measuring the amount of cellobiose produced over time using using the HPAEC-PAD method described above. The initial velocities of each enzyme were plotted against the substrate concentrations, and the kinetic parameters were estimated by fitting the data to the Michaelis-Menten equation using the software GraphPad Prism 5.01 (GraphPad, San Diego, CA).

### Determination of cellobiose phosphorylase activity

To determine whether Ra2122 and Ra2664 function as cellobiose phosphorylases, each protein (5 μM) was incubated with the substrate (10% w/v) and 10 mM sodium phosphate in citrate buffer (pH 6.5) at 37 °C for 30 min. The reaction was stopped by addition of 100 μl of 4 M Tris-HCl. The products were analyzed using HPAEC-PAD. Products were identified by comparing of retention times to known cello-oligosaccharides and glucose 1-phosphate.

### Synergistic activity of cellobiohydrolase (Ra3055), endoglucanase (Ra0903) and cellobiose phosphorylase (Ra2122) on cellulosic polysaccharides

To determine the synergistic activity of cellulose degrading enzymes in *R. albus* 8, a combination of the three enzymes (Ra0903, Ra3055 and Ra2122) were analyzed for their release of end products from different cellulosic substrates. All combinations of Ra3055, Ra0903, and Ra2122 at final concentrations of 0.5 μM each were incubated with different cellulosic substrates (PASC, Avicel and pretreated sugarcane bagasse) (0.5% w/v) in sodium phosphate buffer (pH 6.5) at 37 °C for 16 hours. The hydrolytic reaction was stopped by heating at 99 °C for 10 minutes. The soluble fraction was separated by centrifugation at 25,000 × g for 10 minutes and the released sugars were analyzed using high performance anion exchange chromatography or HPAEC-PAD as described above. Products were identified by comparing their retention time with the retention time of known oligosaccharides. The concentration of the released sugars was quantified by comparison of the peak area to a standard curve created using known concentrations of glucose and cellobiose.

### *In vivo* expression analysis of cellobiohydrolases and cellobiose phosphorylases

The total RNA was extracted from *R. albus* 8 cells grown in a defined medium containing either cellobiose or phosphoric acid swollen cellulose (PASC). The cells were harvested at mid-log phase by combining the culture with 2 volumes of RNAprotect^®^ bacterial reagent (Qiagen), followed by centrifugation at 13,000 × g for 10 min at room temperature. The cell pellets were stored at −80 °C until RNA extraction. In the subsequent steps, the cell pellets were treated with lysis buffer (200 U/ml of mutanolysin, 150 μg/ml of proteinase K, 25 mM EDTA, and 0.5% SDS) for 30 minute at 55 °C. The total RNA was extracted with the RNeasy mini kit (Qiagen) with the optional on-column DNase treatment step. Then, the total RNA was eluted with DEPC-treated nuclease-free water and bacterial ribosomal RNAs were removed with the MicrobExpress kit (LifeTechnologies). The enriched mRNA fraction was converted to RNA-Seq libraries using the TruSeq Stranded RNA Sample Prep kit from Illumina. The expression of cellobiohydrolases and cellobiose phosphorylases were analyzed using CLC Genomics Workbench version 5.5.1 (CLC Bio-Qiagen, Aarhus, Denmark). The genomic sequence of *R. albus* 8 (NZ_ADKM020000001 to NZ_ADKM02000136) was used as the reference genome, and the reads were mapped onto the reference sequences using the CLC software. Reads were only assembled if the fraction of the read that aligned with the reference genome was greater than 0.9 and if the read matched other regions of the reference genome at less than 10 nucleotide positions.

## Additional Information

**How to cite this article**: Devendran, S. *et al.* Multiple cellobiohydrolases and cellobiose phosphorylases cooperate in the ruminal bacterium *Ruminococcus albus* 8 to degrade cellooligosaccharides. *Sci. Rep.* **6**, 35342; doi: 10.1038/srep35342 (2016).

## Supplementary Material

Supplementary Information

## Figures and Tables

**Table 1:** Enzyme kinetics analysis for wild-type and truncational variants of cellobiohydrolase Ra3055.

Ra3055	*k*_cat_ (s^−1^)	*K*_m_ (mM)	*k*_cat_/*K*_m_ (mM^−1^.s^−1^)
WT	71.5 ± 3.6	2.9 ± 0.2	27.1 ± 4.7
TM 1	114.6 ± 7.4	4.5 ± 0.3	32.5 ± 7.1
TM 2	109.7 ± 12.9	5.6 ± 0.9	19.6 ± 1.4
TM 3	97.3 ± 2.4	2.9 ± 0.1	33.8 ± 1.1
TM 4	1.5 ± 0.3	7.9 ± 3.6	0.23 ± 0.1

Each enzyme (0.05 μM) was incubated with varying concentrations of cellotetraose (0.01 mM to 30 mM). The velocity of each reaction was determined by measuring the amount of cellobiose produced over time using HPAEC-PAD. Reaction velocities were plotted against cellotetraose concentrations and where applicable fitted to the Michaelis-Menten equation.
