# Supplementary material for: Multiple cellobiohydrolases and cellobiose phosphorylases cooperate in the ruminal bacterium Ruminococcus albus 8 to degrade cellooligosaccharides
Source: Sci Rep. 2016 Oct 17;6:35342. doi: 10.1038/srep35342 (PMC5066209; doi:10.1038/srep35342)
Supplement: Supplementary Information [file srep35342-s1.pdf]

Supplementary information

Multiple cellobiohydrolases and cellobiose phosphorylases cooperate in the ruminal bacterium *Ruminococcus albus* 8 to degrade cellooligosaccharides

Saravanan Devendran[1,2], Ahmed M. Abdel-Hamid[1,2], Anton F. Evans[1,2,3], Michael Iakiviak[4], In Hyuk Kwon[4], Roderick I. Mackie[1,2,3, 4] and Isaac Cann[1,2,3,4,5]

Energy Biosciences Institute,[1] Carl R. Woese Institute for Genomic Biology,[2] School of Molecular and Cellular Biology,[3] Department of Animal Sciences,[4] and Department of Microbiology,[5] University of Illinois at Urbana-Champaign, Urbana, Illinois 61801, USA

Supplementary Tables

SI Table 1: Primers used in cloning of *R. albus* 8 putative cellobiohydrolases, cellobiose phosphorylase, truncational mutants of Ra3055.

Gene	Forward primer	Reverse primer
Ra1589	5'-gacgacgacaagatggaagacgaacagatacttatccg-3'	5'-gaggagaagcccggtcactttatagtaacagtacaagcacgtttgatag-3'
Ra1743	5'-gacgacgacaagatgggtcagcagttgggg-3'	5'-gaggagaagcccggtttatttatcgtcgccgttattgccgt-3'
Ra3055	5'-gacgacgacaagatgggtcagcagctcggtcaga-3'	5'-gaggagaagcccggtttacttaactttaacggtaactacagagttcttca-3'
Ra2122	5'-gacgacgacaagatgaaattcggtttttcgatgacgctaa-3'	5'-gaggagaagcccggttcagcccattactacttcgacttcg-3'
Ra2664	5'-gacgacgacaagatgcagtatggttattttgacctgaaaaac-3'	5'-gaggagaagcccggttcagcccatcacaacagtgatattgg-3'
TM1	5'-gacgacgacaagatggagatgggtgtcgtaagacctgag-3'	5'-gaggagaagcccggtttacttaactttaacggtaactacagagttcttca-3'
TM2	5'-gacgacgacaagatgggtcagcagctcggtcaga-3'	5'-gaggagaagcccggtttagttggtcatccaaaccagaggtg-3'
TM3	5'-gacgacgacaagatggagatgggtgtcgtaagacctgag-3'	5'-gaggagaagcccggtttagttggtcatccaaaccagaggtg-3'

TM4	5'-gacgacgacaagatggtttacaacacacagctgctgaagg-3'
	5'-gaggagaagcccggtttagttggtcatccaaaccagaggtg-3'
GH9 E882A	5'-cttggtctgtaaacgcagtaactatcaactgg'3'
	5'-ccagttgatagttactgcgtttacagaccaag-3'

SI Table 2: Binding constant of both WT and truncational mutants of cellobiohydrolase

	N	K (M^{-1})	ΔH (cal/mole)	ΔS (cal/mole/deg)
Ra3055 WT	0.69±0.89	2.94E4±7.38E3	-5.17E4±8.24E4	-153
TM1	0.82±0.019	6.38E5±1.57E5	-5.322E3±180.8	8.71
TM2	1.03±0.48	3.49E4±4.59E3	-3.58E4±2.32E4	-99.4
TM3	0.93±0.01	5.06E5±4.41E4	-3.48E3±44.02	14.4
TM4	NB	NB	NB	NB

(Ra3055) with Cellopentaose

NB – No binding

Supplemental Figures

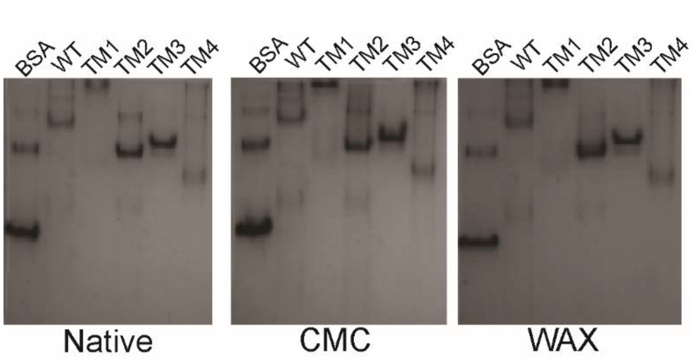

Figure S1 : **Binding of wild-type and truncational variants of the cellobiohydrolase Ra3055 to soluble polysaccharides**. Affinity non-denaturing gel electrophoresis of Ra3055 WT and its truncational variants to the soluble polysaccharides CMC and WAX.

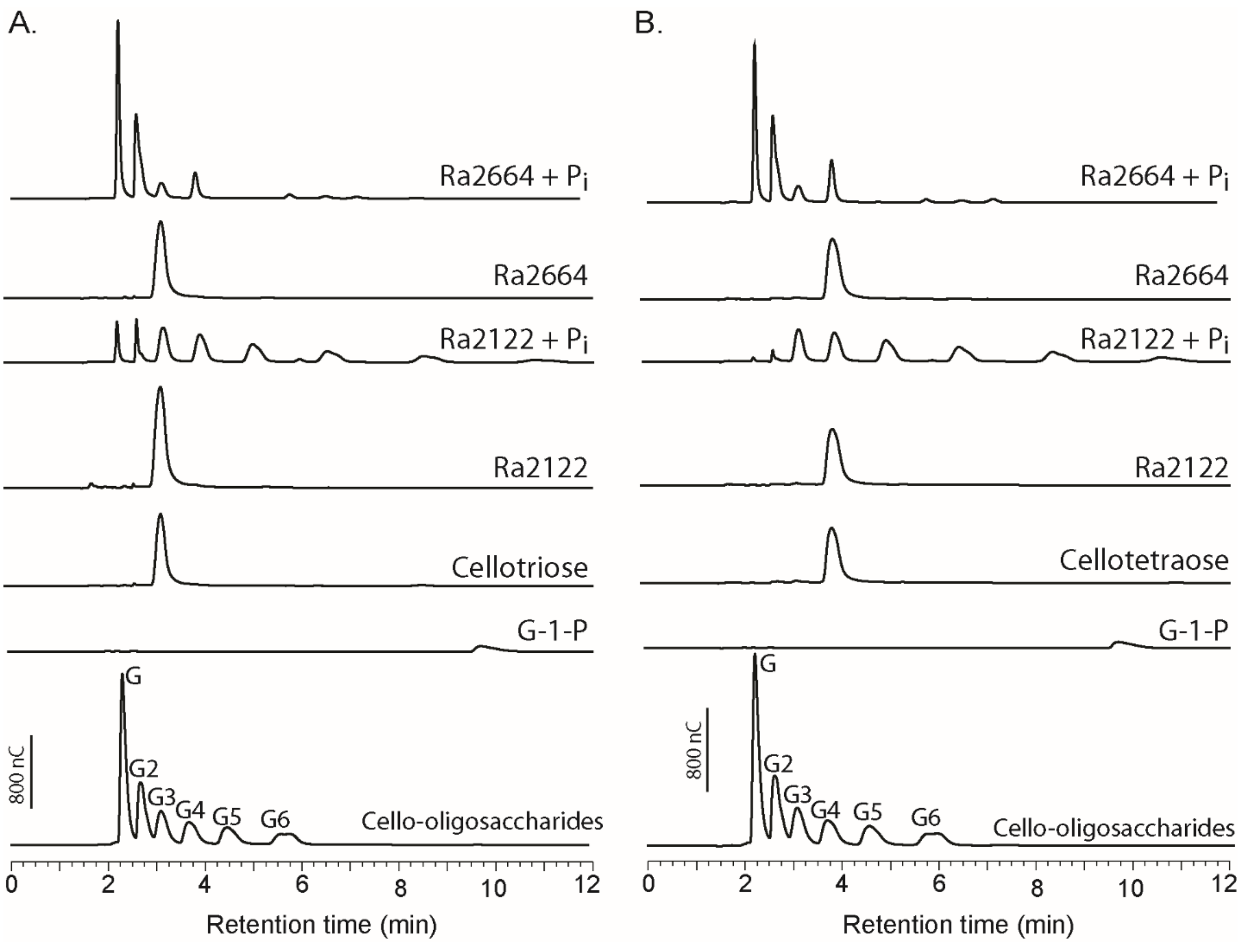

Figure S2: **Activity of putative cellobiose phosphorylase (Ra 2122 and Ra 2664) on cellotriose and cellotetraose.** Each protein (5 μM) was incubated with the substrate (5mM) and sodium phosphate (10 mM) in citrate buffer (pH 6.5) at 37 °C for 30 min. The control reaction mixture was the same except for lacking sodium phosphate. The products were analyzed by HPAEC-PAD. The end products were identified by comparing the retention times to those of known cello-oligosaccharides and glucose 1-phosphate.
